# “Success Depends on Your Backbone”—About the Use of Polymers as Essential Materials Forming Orodispersible Films

**DOI:** 10.3390/ma14174872

**Published:** 2021-08-27

**Authors:** Katarzyna Olechno, Anna Basa, Katarzyna Winnicka

**Affiliations:** 1 Department of Pharmaceutical Technology, Medical University of Bialystok, Mickiewicza 2c, 15-222 Bialystok, Poland; 2 Department of Physical Chemistry, Faculty of Chemistry, University of Bialystok, Ciolkowskiego 1K, 15-245 Bialystok, Poland; abasa@uwb.edu.pl

**Keywords:** film-forming polymers, polymeric materials, pharmaceutical excipients, orodispersible films

## Abstract

Polymers constitute a group of materials having a wide-ranging impact on modern pharmaceutical technology. Polymeric components provide the foundation for the advancement of novel drug delivery platforms, inter alia orodispersible films. Orodispersible films are thin, polymeric scraps intended to dissolve quickly when put on the tongue, allowing them to be easily swallowed without the necessity of drinking water, thus eliminating the risk of choking, which is of great importance in the case of pediatric and geriatric patients. Polymers are essential excipients in designing orodispersible films, as they constitute the backbone of these drug dosage form. The type of polymer is of significant importance in obtaining the formulation of the desired quality. The polymers employed to produce orodispersible films must meet particular requirements due to their oral administration and have to provide adequate surface texture, film thickness, mechanical attributes, tensile and folding strength as well as relevant disintegration time and drug release to obtain the final product characterized by optimal pharmaceutical features. A variety of natural and synthetic polymers currently utilized in manufacturing of orodispersible films might be used alone or in a blend. The goal of the present manuscript was to present a review about polymers utilized in designing oral-dissolving films.

## 1. Introduction

Among the many different routes of drug application (the Food and Drug Administration (FDA) distinguishes about 100 of them), the most preferred and the least invasive is the oral route [1]. Despite many advantages of using conventional solid, oral, drug dosage forms (capsules, tablets), their application might be problematic, particularly for patients facing swallowing problems (pediatric and geriatric) or suffering from depression, schizophrenia, or dementia [2,3,4,5,6]. A modern alternative to traditional solid drug dosage forms are formulations enabling flexible applications both for pediatric patients as well as adults, namely, orodispersible dosage forms that are swiftly disintegrated in the mouth. Disintegration should occur as a result of contact with saliva, forming a suspension, solution, or soft paste that should provide an acceptable mouth feel and easy swallowing. Their use eliminates the possibility of choking that might arise from taking traditional tablets or capsules [3,4,5,6,7,8,9,10].

Orodispersible films (ODFs) represent an innovative system of oral administration of medicines in the form of a thin, polymeric, oral matrix having a thickness in the range from 12 to 100 µm and surface of 2 to 8 cm^2^ [5,6,7,9,11], which should disintegrate within 30 s (for orodispersible tablets, according to FDA and the United States Pharmacopoeia (USP) guidelines, because there are no explicit directives that specify a precise disintegration time particularly for ODFs) [12,13]. Oral films tend to be a convenient drug dosage form, enabling improved dosing accuracy as a single ODF is produced to provide a specified dose of the drug. The ODFs’ production is carried out using the following methods: solvent casting and evaporation, hot melt extrusion, or electrospinning. The main excipients used in their development are film-forming polymers, which seem to be a backbone of the film; however, additional excipients are necessary in order to obtain a complete product with optimal properties, namely, plasticizers, taste-masking agents, sweeteners, surfactants, and saliva stimulators [5,9,14,15].

Plasticizers are an important group of excipients used in the development of ODFs, usually adding up to 20% by weight of the dry polymeric powder. Examples of plasticizers are as follows: glycerol, propylene glycol, mannitol, sorbitol, diethyl phthalate, tributyl and triethyl citrate, macrogol, or esters of citric acid [5,6,14,15,16]. Their main role is to counteract crushing; decrease the glass transition temperature (Tg) of the polymeric material; enhance the elasticity, mechanical features, tensile strength, elongation percentage of the polymeric material; and influence the solubility and absorption of the drug dosage form as well as diminish the bitter taste of a drug. Due to the hydrogen-bonding capabilities of plasticizers (e.g., propylene glycol and glycerin), the inclusion of appropriate quantities of those excipients may enhance the flexibility of ODFs by facilitating the interaction of hydrogen bonding between polymer chain functional groups and the plasticizing agent, leading to the reduction of a polymer–polymer interaction. Several reports consider the impact of plasticizers on the ODFs’ characteristics; therefore, the selection of a suitable one or their blend is a relevant factor. For instance, polyethylene glycol with citric acid esters should not be applied for plasticizing maltodextrin ODFs because of their non-mixability [14]. An increased content of glycerol or propylene glycol in maltodextrin-based ODFs results in a reduced modulus of elasticity and elongation at the break. The concentration of these excipients in amounts greater than 18% (*w/w*) affected the ropiness of ODFs. In vivo organoleptic investigations showed that films made of hypromellose (Pharmacoat 606) and plasticized with polyethylene glycol or a mixture of glycerol and polyethylene glycol were of unsavory taste, while ODFs made of glycerol alone had a more favorable taste than films with propylene glycol. Glycerol ensures improved plasticity of films formed utilizing polyvinyl alcohol, while diethylene glycol might be utilized for ODFs composed of either HPMC or PVA [15,16,17].

Examples of surfactants that improve spreading of the casted solution and wetting by saliva in the mouth, utilized in manufacturing of ODFs, are polysorbate, poloxamer 407, sodium lauryl sulfate, and examples of sweetening/taste-masking agents are flavored essences, sorbitol, mannitol, glucose, aspartame, fructose, sucralose, cyclamate, sucrose, maltose, oleoresins, ribose, thaumatin I and II. As dryness in the oral cavity delays the rapid dissolution of ODFs, it is vital to add salivatory secretion stimulants in order to increase the rate of saliva production. For this purpose, mainly organic acids are used: malic, ascorbic, tartaric, or lactic acid [5,9,15,16].

Polymers are considered as excipients, but they have grown to be a key element in designing of the films, being responsible for a number of their properties. Thus, comprehension of polymers’ physicochemical characteristics and rheology is essential to optimize their utility in ODFs’ preparation. It is necessary to consider a choice of the suitable ones to produce the film, ensuring the most advantageous therapeutic result [5,9,15,16]; hence, owing to the availability of polymers with different characteristics, it is easy to create a film with the desired properties. Polymers constitute about 40–50% of film mass. Their characteristics relate to their molecular weight (MW): Typically, low-molecular-mass polymers dissolve more quickly, while a higher molecular mass implies superior mechanical features. Therefore, if the polymeric material fails to meet the requirements of a specific applicability, its features might be modified. For this reason, the polymers can be physically mixed or chemically reacted [5,9,15,16]. The optimal characteristics of polymers used in designing of ODFs include good spreadability, viscoelasticity, biocompatibility, and compliance with a drug. The selection of those excipients has to be guided by the desired properties of the ODF such as strength of the film, pliability, dissolution rate and moisture content, non-toxicity, capacity of immediate disintegration upon contact with the saliva, sufficient mechanical properties (tensile strength, folding endurance, tear resistance), relevant elasticity, and a structure of ODFs. The mechanical features of the films depend strongly upon the Tg of the cross-linked polymer and upon the degree of cross-linking. It is also of great importance that the substances should not adversely hinder the release of the medicinal substances. In addition, the polymers have to be of good durability and cannot cause secondary bacterial infections in the oral cavity. Whereas ODFs have to dissolve in the mouth as quickly as possible, their mechanical features have to be sufficient for manipulating packaging and storing [5,6,7,8,9,10,15,16]. Thus, rather than a film based on a single polymer, it is preferred to use a mixture of polymers to enhance and optimize the overall characteristics of the ODFs [5,7,9].

The viscosity of the polymer solution is crucial for ODFs’ production and drug release from the designed formulation. The polymeric materials that create a thick gel layer of high viscosity upon wetting will result in slower release compared to polymers of low or intermediate viscosity. On the other hand, solutions having an insufficient viscosity are found to be difficult to cast and obtained films are difficult to handle due to air bubble entrapment. Various viscosity limits are reported in the literature, but these cannot be used as overall recommendations as the required viscosity level is highly dependent on the manufacturing method and equipment used. It is essential to keep in mind that viscosity depends not merely on the quantity of polymer used, but also on its type; therefore, the individual limits should be assessed for each polymer or combination of polymers. A considerable difficulty in the designing of ODFs is to predetermine the optimum viscosity range of the casting compounds. Furthermore, their viscosity, however highly influential, is not the single parameter that affects the behavior of the mass in ODFs’ development, as other factors, associated with the structure of the polymer chains, the interactions of the ingredients, the nature of the solvent or the surface tension, are also influential. Further variations in the characteristics of the casting mass are caused by the presence of the drug. In a suspension-type system formulated by insoluble drugs, further difficulties occur in relation to adequate viscosity adjustment so that sedimentation of particles in the film-casting process does not proceed too rapidly. The viscosity of the casting solution also needs to be chosen depending on the height of the casting gap, with the principle that the thicker the layer of solution applied, the higher viscosity is demanded [18,19,20].

Primarily, water-soluble polymers (either natural, semisynthetic, or synthetic) are employed in formulation of ODFs to obtain a thin matrix characterized by rapid disintegration time, good mechanical properties, and organoleptic sensations. Commonly utilized ones include hydrophilic cellulose derivatives (hydroxypropyl methylcellulose (HPMC), hydroxypropyl cellulose (HPC), polyvinyl alcohol (PVA), polyvinylpyrrolidone (PVP), or polyethylene oxide (PEO)). Recently, the polymers of natural origin, chiefly polysaccharides, have received significant interest in the pharmaceutical industry on account of being safe, biodegradable, and biocompatible. In this regard, either maltodextrin, chitosan, or pullulan is employed. Hydrophilic materials are the leading preference for preparing the ODFs, so that the drug dosage form can dissolve easily and softly in the mouth. Hydrophobic polymers such as methacrylic acid copolymers or polyvinyl acetate (PVAc) are also used, although less frequently [5,9,15,16,18,19,20]. It should be emphasized that all the above mentioned polymers have been proven safe for human use [21,22].

## 2. Hydrophilic Polymeric Materials

### 2.1. Natural Polymers

#### 2.1.1. Cellulose and Cellulose Derivatives 

**Figure 1:**
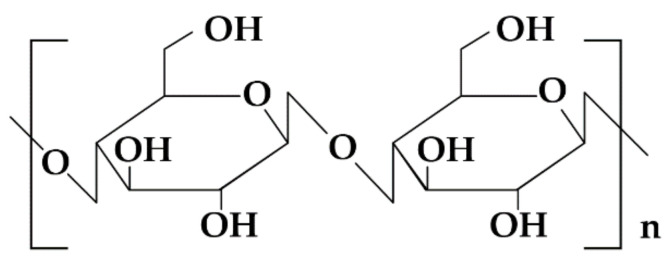
Schematic illustration of cellulose structure.

Cellulose is a commonly found polysaccharide with formula (C_6_H_10_O_5_)n, consisting of linear chains formed by β-D-glucose units connected by β-1,4-glycosidic bonds (Figure 1). The basic repeating unit in the cellulose chain is the disaccharide—cellobiose. The polymer is insoluble in water and indigestible by the human organism. Cellulose fibers remain undissolved both in hot or cold water as a result of presence of strong intramolecular hydrogen bonds in their structure. However, modified derivatives have found a significant range of applications in the pharmaceutical technology—cellulose is processed into cellulose esters or ethers which become water-soluble to increase water retention capacity and film forming properties. Substitution of functional groups results in various attributes of cellulose derivatives. They vary from each other in terms of the kind of substituents, substitution level, molecular weight (resulting in viscosity of a solution), and particles size (Table 1) [7,15,16,18,19,20]. The obvious benefit of cellulose derivatives is that they are biocompatible and thus can be applied for pharmaceutical use—they are deemed to be safe excipient (Generally Regarding as Safe, GRAS) included in FDA list and approved for use in children pharmacotherapy [21]. Hydroxypropyl methylcellulose (HPMC), sodium salt of carboxymethylcellulose (NaCMC) and methylcellulose (MC) are mentioned in Safety and Toxicity of Excipients for Paediatrics (STEP) database [22].

**Figure 2:**
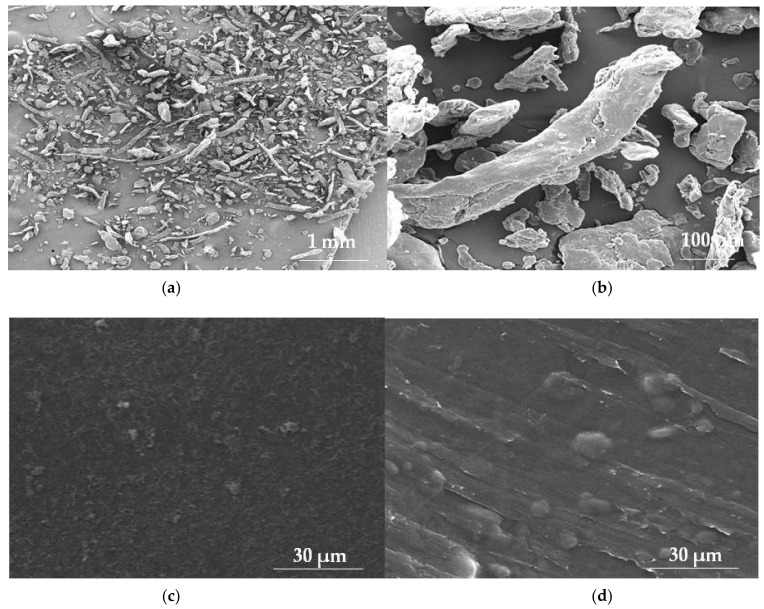
SEM picture of HPMC (Pharmacoat 606) powder under magnification (**a**) 100×, (**b**) 1000× and ODFs’ piece made of HPMC (Pharmacoat 606) placebo under magnification, (**c**) 5000× and with incorporated drug under magnification, (**d**) 5000× (photographs of the authors made with Inspect™S50, FEI Company, Hillsboro, OR, USA).

Among all cellulose derivatives, the most frequently and widely employed in the field of manufacturing of ODFs is HPMC (Figure 2).

HPMC consists of partially O-methylated and O-(2-hydroxypropylated) cellulose, and is accessible in a number of types, differing in molecular weight and number of substituent groups in the anhydroglucose units. The mean amount of methoxy and hydroxypropoxy groups bonded to the ring is expressed as the DS and significantly affects the properties of the oral film, especially in relation to the release of a drug and mechanical features. The hydroxypropoxy group —OCH_2_CH(OH)CH_3_ is a hydrophilic group devoted to the hydration rate, while the methoxy group is considerably more hydrophobic. This feature is considered to be essential for dissolution of drugs and their release profile from ODF. In general, HPMC grades with high viscosity and methoxyl content contribute to obtain more robust, rigid, and stretchable films. Higher viscosity is connected with greater branching and/or higher MW related to physical entanglement due to longer chains. This effect might raise the input strength necessary to break the native chain interactions, resulting in higher tensile strength of the film. In contrast, higher degrees of methoxyl substitution may result in an entrenching effect on HPMC chains due to their larger size relative to the primary hydroxyl groups, which may also lead to high tensile strength. However, it should be emphasized that the degree of methoxyl substitution does not considerably influence the Young’s modulus (the parameter that determines the stiffness of ODF and describes the characteristics of a material with respect to its linear deformation under stresses that occurs in the elastic-deformation range). Regarding the thermal properties of HPMC films, the polarity of the polymer chains resulting from the methoxyl content impacts the Tg. HPMC varieties with less methoxyl groups are characterized by lower polarity, which reduces the free area between polymer chains. The growing distance between the polymer chains results in intensified secondary interactions between the chains, which enhances the energy needed to translocate the chains [18,19,23].

A number of HPMC grades are available for preparation of ODFs and their choice depends on the targeted product features. Currently, the European Pharmacopoeia (Eu. Ph.) and USP define various types of HPMC substituents using a four-digit number, HPMC 1828, HPMC 2208, HPMC 2906, and HPMC 2910, in which the initial two digits relate to the approximate percentage of a more methoxy group (-OCH_3_) and the latter two relate to the approximate percentage of a more hydroxypropoxy group (Table 2) [11,13].

Such variations in the ratios of methoxy and hydroxypropoxy substituents and molecular weight influence their features such as organic thermal gelation temperature in aqueous solution, swelling, solubility, drug release rate, and diffusion. Practically, basic hypromelloses are labelled as type 2208—K, 2910—E. A suffix is additionally given to designate the viscosity of the polymer solution, which corresponds to the molecular weight of the HPMC. It is measured in a 2% aqueous solution (*w/w*) at 20 °C and marked as C (multiplier of 100) or M (multiplier of 1000). The last suffix is needed to verify the grade of product, such as premium (P), low viscosity (LV), controlled release (CR), granular (G), direct compression (DC), surface treated (S), or food grade (FG). The polymeric material is traded under various brand names, for instance, Metolose and Pharmacoat (ShinEtsu Chemical Company, Tokyo, Japan), Methocel (Dow Chemical Company, Midland, MI, USA), and Benecel (Ashland, Rotterdam, The Netherlands) [23,24,25,26].

Basically, type E rather than K is utilized in preparation of the ODFs. Especially, grades E3, E5, and E15 are widely utilized, particularly with regard to their low viscosity and optimal Tg, respectively 160 °C, 170 °C, and 175 °C. The major distinction among these grades is the length of the polymer chain, which is related to the increase in MW of HPMC (E3 < E5 < E15 < E50). The maximum puncture strength increases with MW E3 < E5 < E15 < E50. However, it is stated that E3 and E5 may result in thin, brittle, and non-peelable films. Such features might be improved with increasing concentration of the polymers, but the films are still described as tacky; hence, a blend of various HPMC grades is favored, especially those with higher MW. For instance, E15 has beneficial film-former properties (in terms of transparency and flexibility when mixed with PVA, PVP, microcrystalline cellulose, Eudragit E PO, or maltodextrins plasticized by PEG 400 or glycerol). However, HPMC–maltodextrin mixtures, with higher maltodextrin content, produce thin, fast disintegrating, and palatable films, characterized by an appropriate degree of sweetness. The temperature of degradation of HPMC is in the range from 200 °C to 250 °C (depending on side substitutions of the polymer chain). Considering these characteristics, including high Tg and low temperature of polymer degradation, it should be highlighted that most of the available HPMC grades are not stable during heat treatment [18,23,27]. However, a new product has appeared on the market of pharmaceutical raw materials, Affinisol (DuPont, Wilmington, DE, USA), which provides improved solubility for formulating drugs of low solubility via thermally demanding processes such as hot melt extrusion [28]. It was noted that as the density of HPMC decreases, the particle size and extrinsic viscosity of the solution increases. These variations have a tendency to change the thickness of the film and reduce the strength of the HPMC film, so those of higher density have a stronger and sturdier structure than those of lower density. The density of HPMC is about 1002.5–1012.9 kg/m^3^. Pure HPMC film features a smooth, homogeneous, and uniform matrix. The structure of the HPMC film is greatly affected by the quality of the bonds in the film matrix, which are carried out throughout the drying process [29,30,31].

The literature provides a number of experiments that led to the creation of films based on HPMC. The following polymers were compared in the research to obtain ODFs containing loratadine hydrochloride by solvent-casting method: HPMC (Pharmacoat 606) in concentration of 15% and combination of PVA (7.5%) and PVP (7.5%). All formulations were plasticized with 3% polyoxyethylen glycol (PEG). It has been established that 15% solution of HPMC and a 15% solution of a mixture of PVA05, PVA40, and PVP in a ratio of 2.5:5.0:7.5 are optimal for incorporation of the drug. In the initial trials it was determined that films with high PVP content tended to be too brittle and a higher PVA ratio resulted in both too brittle and too flexible formulations. The mixture of PVP and PVA in the selected proportion led to an appropriate flexibility of the films supplied by PVA and a reduction in disintegration time because of the content of PVP. It was also significant that at the tested polymer concentration in the casting solution, i.e., 15% (*w/w*), compositions containing AP05:AP40:PVP in a ratio of 5:2.5:7.5 or 4:3.5:7.5 were not viscous enough. The viscosities of the HPMC and PVA/PVP solutions chosen for subsequent investigation were 1.9 and 0.76 Pa∙s, respectively. However, such solutions did not enable the preparation of placebo films (deprived of a drug) with uniform thickness and smooth surface from a 1000-µm gap, but using a smaller one (800 µm and less) resulted in proper film formation. On the other hand, the higher viscosity of the solutions was disadvantageous in terms of a venting problem and less uniform mass distribution on the plate [32]. The viscosity of the proposed compositions was also reported to yield a homogeneous drug suspension without rapid sedimentation, which is consistent with Woertz et al., who recommended concentrations of various cellulose derivatives (HPMC—Pharmacoat 606 and HPC—Klucel JXF 140 kDa) ranging from 7.5% to 18%, corresponding to viscosities from 0.5 to 14 Pa∙s for ODFs’ preparation [33]. However, in comparison, films prepared with HPMC exhibit better stability in the aspect of mechanical features and improved homogeneity of loratadine particles along with dosing accuracy while maintaining appropriate physical characteristics. Additionally, HPMC films, regardless of the drug amount, fulfilled the pharmacopeial standard requirements concerning the homogeneity of the medicine concentration, while AP/PVP films were too flexible and uniformity of the drug was not achieved [32]. The aim of another study was to examine the influence of the composition of various viscosity classes of HPMC (E3, E5, and E15) and plasticizers (PEG 400 and glycerol) to prepare zolmitriptan ODFs [34]. All prepared films were translucent and clear and did not show any recrystallization. Polymers’ viscosity affected the increase of tensile strength: about a 2.63-fold rise for HPMC E15 when comparing to E3 (while the increase in film thickness made the formulations were fragile with low tensile strength). Comparable findings were noted for percentage elongation and fold endurance of ODFs. In vitro studies on drug release indicated that higher film thickness and polymer viscosity delayed the disintegration of the films and, consequently, the release of the drug [34]. HPMC E5 (6%, *w/v*) was chosen to prepare everolimus ODFs plasticized with propylene glycol, PEG400, or glycerol [35]. The formulations with propylene glycol were characterized by lower elongation at the break and a higher elastic modulus than those obtained from PEG 400, pointing to reduced elasticity and flexibility. For glycerin, the folding endurance of the ODFs was below 300 times, indicating low flexibility. Films with lower content of glycerin proved to be overly tacky for handling. Those plasticized with PEG 400 were of better flexibility, with higher elongation and lower elastic modulus. Importantly, it was concluded that, when the concentration ratio of HPMC (E5) and PEG400 was 4:3, ODF exhibited the optimal mechanical features, acceptable thickness, disintegration time, and higher Young’s modulus values, with lower percent elongation [35]. In another work, a full factorial experimental design was used to incorporate piroxicam into ODFs while studying the results of some formulation factors on the characteristics of the resulting films [36]. These factors were the casting solvent (water or acetone/water mixture), the film-forming agent (HPMC K4M or sodium alginate), and the solubilization system (no solubilizer, L-arginine, poloxamer, or L-arginine/poloxamer mixture). Sodium alginate as film-forming agent resulted in formation films with greater particles of drug and slower dissolution. Combined use of L-arginine and poloxamer exhibited superior drug dissolution than using each separately. HPMC was more beneficial compared to sodium alginate in terms of mechanical properties and moisture absorption [36]. HPMC (Pharmacoat 606) and various kinds of HPC (Klucel JXF 140 kDa, Klucel EXF 80 kDa, Klucel LXF 95 kDa) were utilized as film-forming polymers in preparation of ibuprofen and loperamide ODFs. Arabic gum was utilized as thickener in certain formulations and glycerol 85% was employed as plasticizer. However, it was observed that formulations with arabic gum were not advantageous in comparison to formulations without thickener in terms of the sedimentation of particles in the suspension, which was observed (therefore, it was excluded from further testing). The viscosities of polymer solutions were specified in initial studies and were dependent on the fraction of polymer. It was a challenge to determinate a precise viscosity range in which all suspensions resulted in fine film, but a fraction range for each specific polymer was defined based on the results of the viscosity measurements for the placebo solutions. HPC suspensions led to significantly higher viscous suspensions in the same range of polymer fraction than HPMC suspensions, but still provided acceptable films. Suspensions exhibited an increase in viscosity by raising the fraction of HPMC from 12.5% to 17.5%. This range was determined to be adequate for suspensions containing Pharmacoat 606 and 1.5% of loperamide. A range between 10% to 12% HPC (Klucel JXF) was suitable for suspensions containing 0.9% of the drug. Reducing the polymer fraction resulted in insufficient viscosity and, thus, a contraction of the suspension after casting, leading to increased film thickness. A decrease of polymer fraction could also result in significantly longer drying times, which is not desired. An increase of polymer fraction would probably still provide an acceptable film, but a high viscous solution or suspension leads to difficulties in the film-casting process and is, therefore, not favorable. Increasing the polymer fraction resulted in higher viscosity of suspensions containing ibuprofen. The increase was more pronounced when compared to the suspensions with loperamide, perhaps due to the high drug load of the suspensions. Within the HPC types, viscosity decreased in the rank order Kluce JXF > Klucel LXF > Klucel EXF. Klucel LXF shows a viscosity of 75–150 mPa∙s in aqueous solution at 25 °C with a polymer fraction of 5%. Klucel JXF shows higher viscosities of 150–400 mPa∙s under the same conditions. Klucel EXF has the lowest viscosities of 300–600 mPa∙s at 25 °C and a polymer fraction of 10% in aqueous solution [33]. Brniak et al. designed HPMC (in concentration 12.5%, *w/w*)-based ODFs’ films containing prednisolone encapsulated in spray-dried microparticles made of Eudragit E PO with glycerol or PEG 200 as plasticizer. Films containing glycerol at 3.125% or 3.75% concentration were brittle and fragile, while formulations containing PEG 200 were found to be more flexible and resistant. However, ODFs containing PEG 3.75% were too viscous and ductile, so a formulation of 12.5% HPMC and 3.125% PEG was selected as optimal [37]. In another work, HPMC, PVP, pullulan, and HEC were selected as backbones for solid dispersions of tetrabenazine. The influence of the chosen polymeric materials was investigated. Evaluation of the ODFs’ properties for 6 months allowed identifying optimal formulations based on the HMPC and pullulan matrices, while recrystallization of the drug occurred in films made of PVP and HEC, which, as the consequence induced a decrease in drug release evaluated in saliva conditions. HPMC and pullulan maintained tetrabenazine in amorphous state for 6 months [38,39]. Two grades of HPMC (E5 and E15 at concentrations of 10, 12.5, 15, and 20%, *w/v*) were used as the film-forming agents to fabricate phenytoin-loaded ODFs and they were evaluated in terms of extrudability and printability by a syringe extrusion by 3D printing method. Glycerin and propylene glycol were utilized as plasticizers. The diameters of the printed fibers were found to decrease with increasing concentrations of HPMC E5 and HPMC E15. However, it was also observed that the inclusion of the drug affected the viscosity and printability of the prepared dispersion. The formulations prepared from HPMC E15 (12.5%, *w/w*) with phenytoin were not extruded smoothly through the nozzle because of its high viscosity and fast drying of the printing dispersion, while the formulations of HPMC E5 (20%, *w/w*) and type E15 (10%, *w/w*) were effectively extruded through the nozzle. The consistency factor scores of HPMC E5 were found to be lower compared to those made of HPMC E15 at the same concentration, suggesting that HPMC E15 shows non-Newtonian pseudoplastic behavior as well as optimal extrudability properties through the extrusion nozzle. The viscosities of 10, 12.5, 15, and 20% (*w/v*) of HPMC E5 were 0.80 ± 0.04, 1.70 ± 0.03, 3.02 ± 0.18, and 9.84 ± 0.08 Pas and the viscosities of 10 and 12.5% (*w/v*) of HPMC E15 were 8.10 ± 0.51 and 16.85 ± 0.76 Pas, respectively. It was evaluated that higher viscosity value was observed when the drug was incorporated into the formulations. In the case of E5 20% and E15 10% formulations, after phenytoin was incorporated, the viscosities were determined to be 13.39 ± 0.74 and 12.17 ± 0.47 Pas, respectively. Furthermore, the findings demonstrated that, for a lower-molecular-weight polymer (HPMC E5), the higher polymer concentration is necessary to obtain a similar viscosity as the high-molecular-weight polymer (HPMC E15). Then, printed ODFs characterized by optimal mechanical properties and the fastest disintegration time were selected to evaluate their drug content and dissolution profiles. The results showed that ODFs based on HPMC E15 demonstrated superior properties when compared to E5 films. Fast disintegration time in less than 5 s and rapid dissolution (up to 80% of drug was released within 10 min) were demonstrated. In addition, a formulation made of HPMC E15 exhibited drug content uniformity, good mechanical properties, flexibility with low puncture strength, low Young’s modulus, and high elongation, which enables easy handling and application [40].

HPMC of different grades (E3, E5) and NaCMC, all plasticized with PEG 600 and glycerol, were evaluated for the ability of creating ODFs with donepezil. The formulations (F1–F3), with 30% (*w/w*) polymer concentration, had the following composition: F1, HPMC E3; F2, HPMC E5; and F3, HPMC E5 (26%) along with NaCMC (4%). It was assessed that all ODFs dissolved quickly and were readily disintegrated, owing to the hydrophilic nature of polymeric materials used. However, changes in the drug release profiles were noted, which were probably associated with variations in the polymer type: A significant increase was observed for the F3 formulation, which released 93% of the drug under 8 min. The formulation F3 dispersed quickly in the medium and homogenic distribution of drug particles was observed. An addition of NaCMC greatly influenced the percentage of released dose, as the combination of HPMC and NaCMC increased the hydrophilic characteristics of ODFs. Additionally, the formulation F3 exhibited high roughness of the surface and porosity, which might be related to rapid drug-release behavior. Hydrogen-bonding interaction between drug and polymer were revealed by spectral analysis [41].

The following polymers, HPMC E3, Carbomer 947, HPC, HEC, and sodium alginate (SA) in different combinations plasticized with glycerol 85%, were used to prepare bilayered ODFs containing enalapril maleate [42]. It was decided to create double-layered films, considering the possibility of incorporating a higher dose of the drug. The basic solution consisted of HPMC and carbomer formed the first layer of the matrix. It was observed that the appearance of the bilayered ODFs was affected by the sequence in which the layers were added. Moreover, obtained results showed that not every combination of polymers enables obtaining an acceptable film. The ODFs obtained on the basis of two layers of the basic HPMC/carbomer solution were uniform and elastic. Utilization of casting solutions made of HPC or HEC as the second layer and HPMC/carbomer as the first one led to the formation of thin ODFs. The films obtained from SA as the first layer were not feasible, because their surface was very smooth and soft, which rendered them unable to be measured in terms of the mechanical properties because ODFs slipped from the grips of the analyzer. HPC used as the second layer (on HPMC/carbomer one) exposed gaps, which may be generated by irregular evaporation of the solvent and high surface tension of the casting solution. The other drawback of the HPC-based casting solution is the long drying time (about 8 h, instead of the usual 1.5 h). Only a few of the proposed polymeric combinations exhibited a mild to good mouth feel, namely, bilayered films made of HPMC and carbomer, HPMC/carbomer/HPC, and bilayered HPC/HPC. The others were floating in the oral cavity, which is disadvantageous for patient compliance. The combination HPMC/carbomer/HEC led to ODFs with a disintegration time longer than 3 min. [42].

Explored ODFs’ compositions based on cellulose derivatives are shown in Table 3.

#### 2.1.2. Starch and Starch Derivatives

**Figure 3:**
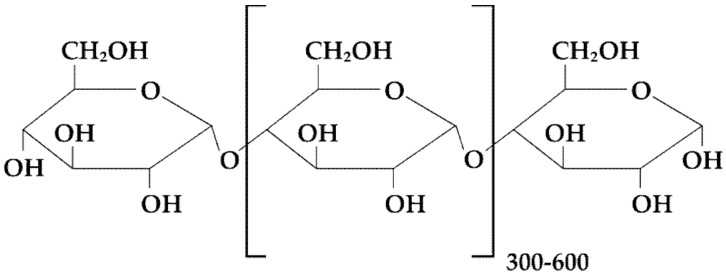
Schematic illustration of starch structure.

Within all naturally occurring biopolymers, starch has been regarded as one of the most prospective polymers due to its widespread availability, biodegradability, and relatively low cost. It consists of two polymers, namely, amylose (an unbranched spiral polymer composed of α-1,4 combined D-glucose monomers) and amylopectin (a highly branched polymer composed of both combined α-1,4 and α-1,6 D-glucose monomers) (Figure 3). A variety of starches are identified for pharmaceutical application. Depending on the plant origin or source, there are various types of starch, e.g., corn, pea, rice, potato, maize, or wheat starches [18,51].

Starch granules consist of three particular regions, which are the amorphous region, the crystalline lamellae, and the amorphous growth ring. Considering the stable semi-crystalline framework of starch, the granules are not dissolved in water at room temperature. Nevertheless, it is subjected to restricted swelling, probably as a result of hydration and swelling of the amorphous regions. It is recognized that amylopectin, because of its highly branched and open structure, enables hydrogen-bonded solvent molecules to access its composition. Therefore, in water or any other solvent capable of forming hydrogen bonds, it undergoes more disruption than amylose, which features a highly packed structure. The interaction of starch granules with hot water is distinct from that of room temperature. Exemplary, when starch granules are heated in excess water above a critical temperature that depends on the type of starch, an irreversible change called gelatinization occurs. Applicability of starch is restricted due to its efficient barrier against low polarity compound and its poor mechanical strength. Plasticizers are generally required for oral films composed of starch to overcome film brittleness. Usually, employed plasticizers for starch films are glycerol and sorbitol. In general, low concentrations and high temperatures are needed to dissolve the starch. Process-related problems also concern the difficulties connected with solubility of native starch in water, owing to its large molecular size and strong hydrogen bonds. Therefore, to surmount this drawback and to enhance the performance of the ODFs, various starch derivatives have been engineered. Exemplary modified starches used in the formulating of ODFs include hydrolyzed starches such as pregelatinized starches, maltodextrins, or pullulan [18,51,52].

**Figure 4:**
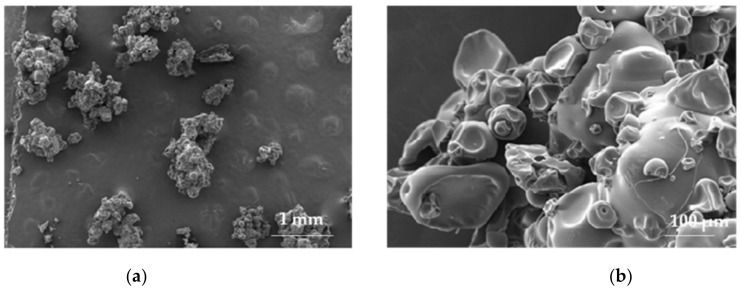

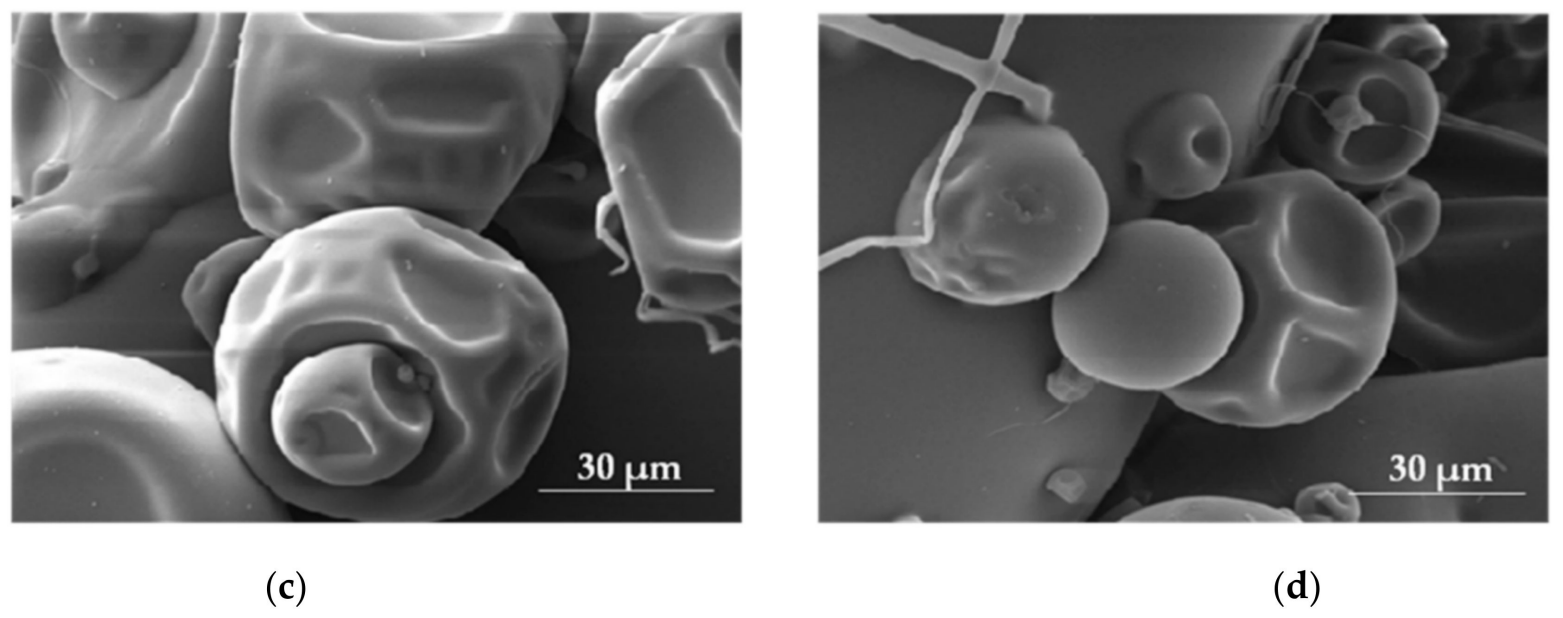
SEM illustration of pregelatinized pea starch (Lycoat RS 720) under magnification: (**a**) 100×, (**b**) 1000×, (**c**,**d**) 5000× (photographs of the authors made with Inspect™S50, FEI Company, Hillsboro, OR, USA).

**Figure 5:**
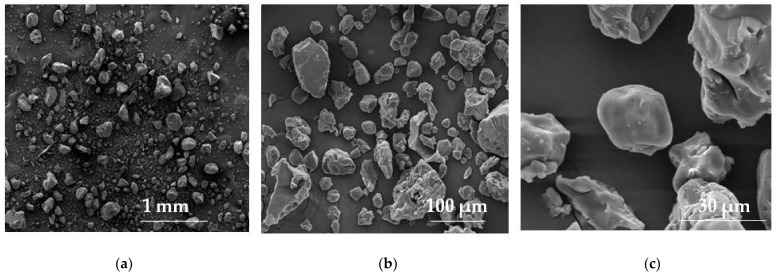
SEM picture of pregelatinized maize starch (StarCap 1500) under magnification: (**a**)100×, (**b**) 1000×, (**c**) 5000× (photographs of the authors made with Inspect™S50, FEI Company, Hillsboro, OR, USA).

Pregelatinized starch is chemically and/or mechanically modified starch, commercially available in fully or partially pregelatinized form. The first one is easily soluble in cold water, while the second one has soluble (gelatinized) but also insoluble fractions. Pregelatinized starch features good film-forming properties, creating transparent, robust, and elastic films using a solution at a concentration of 15% to 20% in the solvent-casting process [18,51]. Lycoat RS 720 is commercially available pregelatinized hydroxypropyl pea starch soluble in cold water [53] (Figure 4 and Figure 5).

An interesting comparative study, including multiple polymers, was carried out by Kathpalia et. al. [54]. The goal of their research was to assess the possibility of creating films from different polymeric materials, modified starch (Lycoat® RS 720), pullulan, HPMC E5, or blend of polyvinyl alcohol (PVA) and polyethylene glycol (PEG), commercially known as Kollicoat IR, containing levocetirizine dihydrochloride, plasticized with various plasticizers, glycerol, propylene glycol, sorbitol, or polyethylene glycol 400 (PEG 400). ODFs made of Lycoat in a concentration of 25% (*w/w*) created products with favorable peelability, whereas at the lower concentration of 10% (*w/w*), no film creation was noted. PG and polysorbate 80 were used as plasticizers. PG in concentration 2% (*w/w*) gave fragile ODFs with low folding strength; therefore, its concentration was raised to 4% (*w/w*), resulting in elastic matrices with optimal folding strength. In both trials, the concentration of polysorbate 80 was constant: 2% (*w/w*). Nevertheless, such composition did not allow the obtained films to disintegrate, so PG was substituted with sorbitol (4%, *w/w*), which shortened the disintegration time. It was later noted that when combining with soya lecithin, polysorbate was needed at a lower concentration. Therefore, 0.1% (*w/w*) polysorbate 80 and 0.8% (*w/w*) soya lecithin were added to promote wetting and disintegration (which occurred within 30 s). Additionally, obtained films were smooth, transparent, and non-tacky, having a thickness of 0.2 mm. Pullulan created a well-peeled film, both at concentrations of 2% (*w/w*) and 5% (*w/w*) with an in vitro disintegration time of less than 30 s; hence, the lower pullulan concentration was selected for further studies. Sorbitol was chosen as a plasticizer, as it provided a satisfying folding endurance. Drug-loaded pullulan films were non-tacky, transparent, and disintegrated within 15 s. The 7% (*w/w*) HPMC E-5 with PG 1% (*w/w*) formed well-peelable, fast-disintegrating (10 s) films. In the case of Kollicaot IR, a concentration of 10% (*w/w*) was chosen, as ODFs made of 5% (*w/w*) softened and at 2% (*w/w*) the matrices were not formed. There was no need to plasticize the films, as Kollicoat IR is composed of 75% polyvinyl alcohol and 25% of PG; therefore, only a wetting agent, polysorbate (0.1%), was applied. Obtained ODFs were non-tacky, flexible, transparent, and disintegrated within 21 s [54].

**Figure 6:**
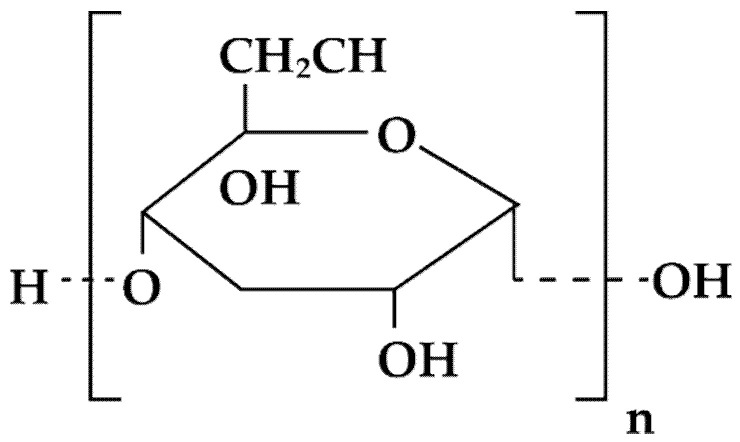
Schematic illustration of maltodextrin structure.

Maltodextrin is an unsweet, water-soluble polysaccharide composed of D-glucose units generally associated with an α (1→4) glycoside bond, combined in chains of variable length, with a dextrose equivalent (DE) below 20, produced by the partial hydrolysis of native starch (Figure 6). It is easily soluble and gets dispersed immediately in water but is slightly soluble to almost insoluble in alcohol. DE value is determined as a yardstick for the total reduction capacity of all sugars contained in the hydrolysate material relative to glucose, which is regarded to be 100. Maltodextrin with low DE values is characterized by high MW, which increases elasticity and prevents cracking. Furthermore, the DE content influences a number of physical and functional features such as solubility and taste. Maltodextrin with DE under 20 is a nutritional saccharide compound of polymers composed of D-glucose units. With higher DE rates, the solubility and hygroscopicity are increased, but the viscosity, the anti-crystallizing power, and the solidification point are decreased. Low DE maltodextrin offers greater viscosity and improved film-forming properties than maltodextrin containing higher DE. It also exhibits improved elasticity and reduced cracking. It was shown that maltodextrins create ODFs featuring good quality with appropriate disintegration time [18,51].

The unique feature about the films crafted from maltodextrins is that they are extremely thin and elegant. In addition, the use of maltodextrin together with microcrystalline cellulose leads to the formation of non-sticky films with a smooth surface [9,18,51]. Maltodextrin (3.5%) along with HPMC E6 (2%) served as an optimal backbone for verapamil ODFs [55]. Cilurzo et al. described the utilization of low DE maltodextrin content as a film-forming polymer for the development of ODFs containing piroxicam. Regardless of the reduced film plasticity with regard to the introduction of the drug in a powder form, the prepared formulation presented optimal elasticity, resistance to elongation, and quick dissolution [14]. The delivery system of melatonin based on the inclusion of solid lipid microparticles into ODFs prepared from MDX plasticized with glycerin, Span 80, and Tween 80 was obtained and characterized by good pharmaceutical properties [56]. The aim of the next research was to design ODFs containing benzydamine hydrochloride. Maltodextrin was employed as film-forming polymer and plasticized with xylitol and sorbitol. The ODFs containing the polymer with lower DE value were of better mechanical features and shorter disintegration time compared to those with higher content. Matrices utilizing xylitol as plasticizer exhibited shorter disintegration time than films containing sorbitol [57]. Cholecalciferol (2000 i.u.) films made of maltodextrin, glycerol, mannitol, polysorbate 80, copovidone, and sodium alginate were obtained by the film-casting method. The use of maltodextrin gave benefits in regard to pliability, physical performance, and stability, and the supplement of alginate improved the physical stability of this mixture [58]. Another study was concerned about evaluation of the applicability of the rice starch (physically modified) obtained by alcoholic-alkaline or planetary ball-milling techniques as a film-forming polymer to obtain ODFs with desloratadine. It was shown that only the utilization of a ball-milled polymer resulted in the development of acceptable films with optimal mechanical features, greater flexible behavior, and disintegration time of about 60 s with more than 95% dissolution within 10 min [59].

**Figure 7:**
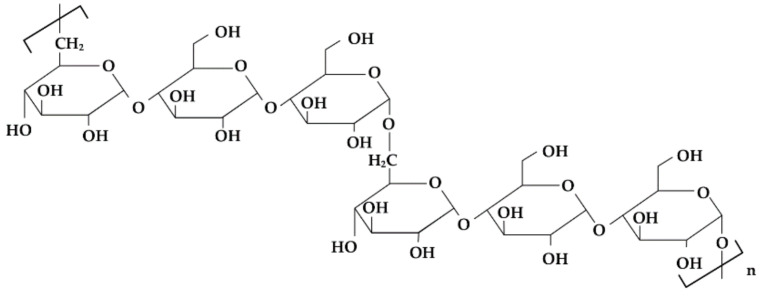
Schematic illustration of pullulan structure.

Pullulan is a natural polysaccharide made by yeast-like fungus *Aureobasidium pullulans*. It is a modified starch consisting of glucose units in maltotriose linked by α(14) glycosidic bonds, whereas further maltotriose units are linked by α(16) glycosidic bonds (Figure 7). Raw polymer is a white to off-white, unflavored, and unscented powder, freely dissolved in hot or cold water, that creates a highly viscous solution at a concentration of 5–25% [18,51,60,61].

Transparent, elastic, and smooth ODFs of great solubility and optimal mechanical strength are obtained from pullulan. An important feature is that pullulan films’ flexibility is accompanied by high tensile strength and stability over a broad temperature spectrum. However, as the substance is sourced from the enzymatic fermentation process of yeast, its low availability (due to complex acquisition) makes it a highly expensive material. Hence, it is typically mixed with alternative polymers that are freely available and less expensive. In general, 50% to 80% of pullulan may be substituted with starch or modified starch without losing its desired performance as a favorable film-former excipient. Sodium alginate or carboxymethylcellulose (CMC) might be employed as well, due to their biocompatibility. Chiefly, the formation of hydrogen bonds between -COO alginate and CMC groups with –OH of pullulan can synergistically improve the material properties of the resulting films. Moreover, pullulan–HPMC blends, with HPMC content above 50%, were also reported and the final polymeric matrix showed better thermal and mechanical properties. The mechanical behavior of pullulan matrices is influenced by the temperature at which it was produced. ODFs formulated at low temperatures are stiffer and more flexible than those made at higher temperatures, which are brittle and do not exhibit pronounced plastic deformation. ODFs made of pullulan are also characterized by a fast disintegration time [9,18,51,60,61]. Recently, the polymer has been used to produce ODFs obtained by the electrospinning method containing isoniazid for treatment of tuberculosis in pediatric patients. The formulations were prepared using pullulan as a base polymer and mixtures of pullulan with polymers such as pectin, sodium caseinate (NaCas), and HPMC (pullulan/HPMC, pullulan/pectin, pullulan/NaCas), maintaining the overall polymer concentration consistent at 19% (*w/v*). For membranes containing HPMC, a greater fiber diameter and a uniform diameter distribution were noted in comparison with pectin and NaCas membranes. The highest viscosity values obtained for HPMC membranes could be ascribed to the existence of a hydroxyl group in their molecule, which enhances the water-binding capacity, resulting in fibers with larger diameters. The incorporation of HPMC and pectin led to the quick disintegration of the membranes in 5 s, while the other formulations were over 15 s. However, the membranes made of a pullulan/HPMC mixture were considered as the best candidate for ODF formulations due to their rapid disintegration in a simulated saliva environment in less than 5 s, allowing convenient administration, as well as the complete release of the drug within 30 s, making the drug easily absorbed in the mouth and throughout the gastrointestinal tract after being swallowed [62].

#### 2.1.3. Sodium Alginate and Chitosan 

**Figure 8:**
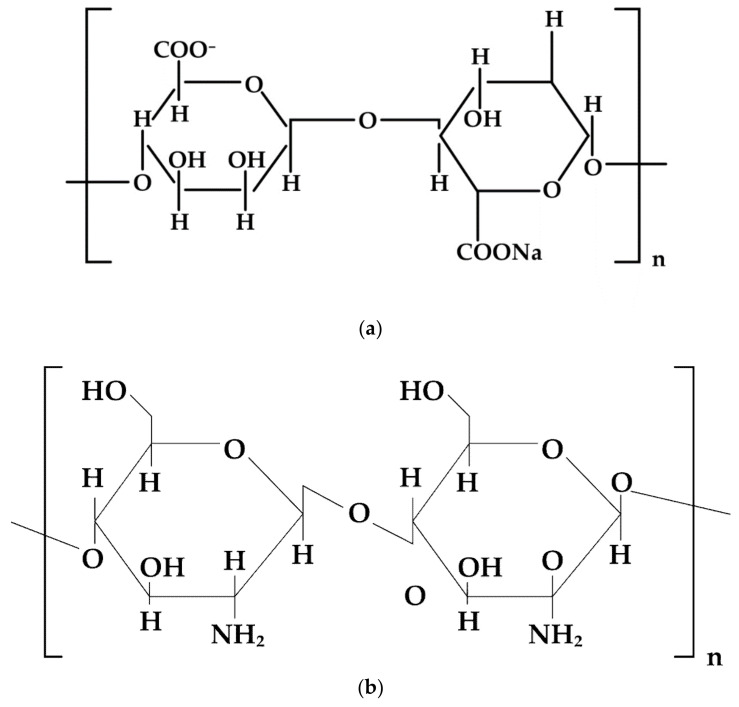
Schematic illustration of (**a**) sodium alginate, (**b**) chitosan chemical structure.

**Figure 9:**
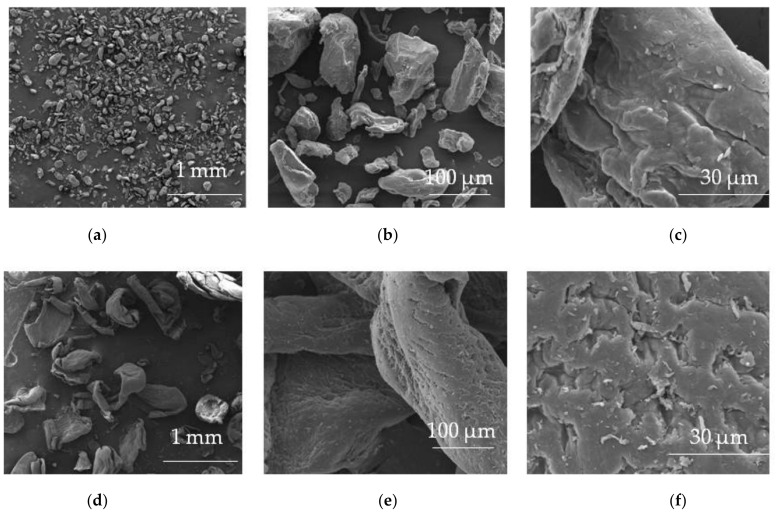
SEM picture of sodium alginate under magnification (**a**) 100×, (**b**) 1000×, and (**c**) 5000× and chitosan particles under magnification (**d**) 100×, (**e**) 1000×, and (**f**) 5000× (photographs of the authors made with Inspect™S50, FEI Company, Hillsboro, OR, USA).

Sodium alginate and chitosan (Figure 8 and Figure 9) are used in the production of ODFs, however, less frequently than commonly utilized HPMC. In Table 4, the characteristics of the polymers and their utilization in ODFs’ technology are briefly described [18,61,63].

An interesting example of novel biopolymers are okra and moringa gum. Natural gums are chemically inert, environmentally friendly, non-toxic, biodegradable, biocompatible, and non-irritating. They also have the advantage of being easy to access and they are cheaper than synthetically modified gum. Okra is typically obtained from *Abelmoschus esculentus *L., a plant extensively farmed in tropical, subtropical, and temperate regions throughout the world. Okra extracts contain acidic polysaccharides consisting of various sugars including galactose, rhamnose, galacturonic acid, galactose, glucose, and glucuronic acid. Moringa gum is obtained from *Moringa oleifera* (a plant native to Western and sub-Himalayan tracts). It is a polyuronide composed of arabinose, galactose, and glucuronic acid in the ratio of 10:7:2, with rhamnose present in trace amounts. Okra and moringa gums have been tested for film-forming properties after blending with pullulan or HPMC to prepare ODFs with citalopram. It was noted that only okra biopolymer might be used in combination with HPMC or pullulan for producing appropriate formulations. Obtained ODFs were smooth, apart from those produced with HPMC and moringa gum combination, which were slightly rough in texture. The disintegration time was less than 30 s and the homogeneity of the drug content was 97.89–102.05% for all formulations. Additionally, the films made of okra gum and HPMC were characterized by superior mechanical properties compared to okra and pullulan blend or moringa gum and HPMC mixture [68,69].

### 2.2. Synthetic Polymeric Materials

#### 2.2.1. Polyvinyl Alcohol (PVA)

**Figure 10:**
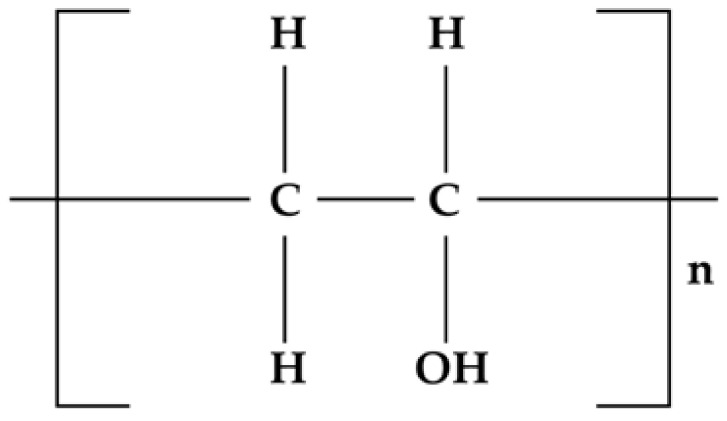
Chemical structure of polyvinyl alcohol.

The polymer is non-toxic (authorized by the FDA for human consumption and considered as GRAS) with high water solubility [21]. The material is obtained by polymerization of vinyl acetate monomer to polyvinyl acetate and subsequent hydrolysis of the acetate groups of polyvinyl acetate with randomly positioned side groups (Figure 10). It occurs as a white to cream-colored granular powder with MW ranking from 20,000 to 200,000. Water represents the most essential solvent for PVA. The solubility of PVA in water is determined by the degree of polymerization, hydrolysis, and solution temperature. Any variations in these parameters influence the degree and nature of hydrogen bonding in aqueous solutions and, thus, the solubility of PVA. PVA has valuable properties including biodegradability, biocompatibility, chemical resistance, and good mechanical properties; therefore, the polymer has found applications in the ODFs’ development, creating films described as very flexible [18,70]. The polymer is a tasteless, odorless, hydrophilic material. In the solution it forms inter- and intramolecular hydrogen bonds between –OH groups available in its monomer units. Water is used as a common solvent for the production of PVA film. Above the glass transition temperature (≈85 °C), fully hydrolyzed PVA is completely soluble in water. The interaction between PVA and water molecules plays an important role in polymer chain dynamics in the solution. With continuous evaporation of water, this dynamic changes in time and the final film is obtained from casting solution [18,71].

PVA-based ODFs plasticized with PEG and glycerin containing salbutamol sulphate were obtained by solvent casting technique. ODFs were transparent with smooth surface, characterized by optimal mechanical properties and demonstrated an immediate release of 90% within the first 2 min. The dissolution profile and physico-mechanical features such as appearance, surface of pH, softness and elasticity were accessed as good [69]. PVA as a backbone of ODFs was utilized to prepare ODFs containing sumatriptan succinate and metoclopramide hydrochloride. The polymer was used in concentrations of 10%, 12.5% and 15% (*w/w*) with variable content of glycerol by solvent casting technique. PVA (15%) with 5% glycerol content showedthe most relevant results. Obtained films were smooth with uniform surface morphology, possessed good tensile strength and disintegration time (ranged from 7.7 to 28 s) and released more than 50% of drug within first 2 min [72]. The aim of the next study was to evaluate utilization of PVA or high metoxypectin (both 1%; *w/w*) to prepare ODF with phenytoin by solvent casting method. From the obtained formulations, these consisting of 1% (*w/w*) PVA, 0.04% (*w/w*) sodium starch glycolate and PEG 400, glycerin and water as cosolvents led to development of promising products. The physical appearance (transparent and colorless ODF with a smooth surface), mechanical strength, drug content and disintegration time were found to be optimal [73].

#### 2.2.2. Polyvinyl Pyrrolidone (Povidone, PVP)

**Figure 11:**
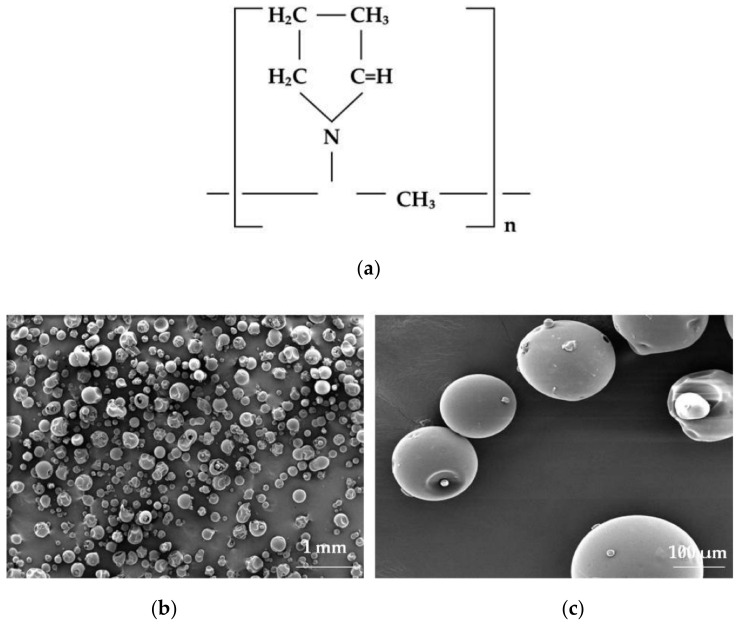
Chemical structure of (**a**) polyvinyl pyrrolidone and SEM picture of polyvinyl pyrrolidone 40 powder under magnification (**b**) 100×, (**c**) 1000× (photographs of the authors made with Inspect™S50, FEI Company, Hillsboro, OR, USA).

Polyvinyl pyrrolidone is a non-ionic, synthetic polymer composed of linear 1-vinyl-2-pyrrolidinone groups (Figure 11) obtained by polymerization of vinylpyrrolidone in isopropanol or water. As the substance is soluble in both aqueous and organic solutions, it offers flexibility in the choice of solvent. PVP is available with various MW (40,000 to 360,000) according to the degree of polymerization. Commercially accessible PVP is classified according to its press K value into four viscosity classes: K-15, K-30, K-60, K-90, having average molecular weight of 10,000, 40,000, 160,000, and 360,000, respectively. PVP appears as a fine, white to creamy-white colored, odorless or almost odorless, hygroscopic powder. Povidones which K-values are equal to or lower than 30 are produced by the spray-drying and occur as spheres. Povidone K-90 and higher K-value povidones are obtained by drum drying and come up as plates. PVP-based dried films are transparent, clear, glossy and hard. It is irrelevant for the appearance of which solvent it was casted from s (water, chloroform, ethylene dichloride or ethanol). Compatible plasticizers can be included with no impact on transparency or glossinesss of the film. Moisture absorbed from the air by PVP may also behave as a plasticizer [18,74].

ODFs with PVP as a polymeric backbone were created with enrofloxacin by solvent-casting method, exhibiting good uniformity and mechanical properties. Their surface pH was 6.8, indicating that these formulations would not cause irritation to the oral mucosa. Average time for the film to disintegrate into completely fine particles was about 1.26 min [75].

#### 2.2.3. Polyethylene Oxide (PEO)

**Figure 12:**
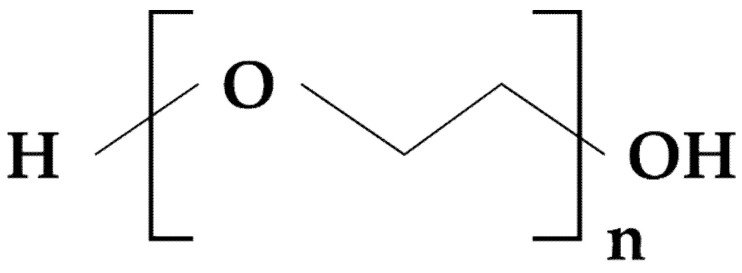
Chemical structure of polyethylene oxide.

Polyethylene oxide is an example of further synthetically derived polymeric material (Figure 12), which found application as a film-forming agent for oral films due to its properties. PEO is a material of high molecular weight with hydrophilic and nonionic nature. It is interesting to note that due to its low Tg (around −67 °C), PEO may be applied as a self-plasticizing polymeric platform, in particular, for MW in the range from ~100 kDa to ~4000 kDa. This property obviates the use of an additional plasticizer in the composition of ODFs, enabling increasing the drug charge, due to lower number of excipients (56% of the film weight). ODFs made of PEO are referred to as films, characterized by optimal tear resistance, fast dissolution rate, and minimal or no curl occurrence. In addition, it was reported that the dissolution time for different PEO grades increases with the molecular weight: N-10 (MW = 100 kDa) < N-80 (MW = 200 kDa) < N-750 (MW = 300 kDa) < WSR 205 (MW = 600 kDa) [18,74]. It was also stated that ODFs made of PEO are characterized by a pleasurable mouth feel, with no sticky sensation or creation of a highly viscous gel. The preferred properties of the achieved oral film may be projected by applying various grades and compositions of PEOs. Accordingly, it is feasible to offset the tear resistance, dissolution rate, and adhesion tendency of film compositions combining PEO having a low MW value from 50% to 75% with PEO having a higher MW value and/or with cellulose derivatives such as HPMC or HPC [18,76].

Olanzapine ODFs were developed from PEO as film-forming polymer with the addition of PVP (Kollidon^®^ VA 64) and poloxamer types 407 and 188 on 3D printer. All of the obtained ODFs were characterized by a uniform drug content, optimal strength, and rapid disintegration time and exhibited good printability. The formulation containing PEO, PVP, and poloxamer 188 indicated the fastest dissolution time and immediate dissolution profiles [77].

## 3. Hydrophobic Polymeric Materials

Most commercially available and laboratory-created oral films are usually made of hydrophilic film-forming polymers. By definition, hydrophilic components possess a greater affinity for water, in comparison to hydrophobic substances. In this regard, the physical, chemical, and microbiological stability of hydrophilic matrices can be severely affected by moisture if they are not protected by appropriate packaging. When directly exposed to regular environmental humidity, these hydrophilic structures can become sticky when the relative humidity is high or brittle when the relative humidity is very low. In either case, it leads to adverse texture and appearance of the obtained product. At the molecular level, small or less perfect crystalline polymer structures can be lost, contributing to lower Tg. In contrast, hydrophobic polymers are known to absorb less water even at high moisture content at room temperature. The hydrophobic nature appears to be incompatible with rapid disintegration in the case of ODFs, so the presence of a channeling agent (like mannitol, microcrystalline cellulose, sodium chloride) by promoting fluid penetration can provide rapid disintegration [7,78]. However, there are not many examples of such formulations in the literature. Musazzi et al. evaluated the feasibility of using Eudragit® S 100 as a polymer for potential use in the formation of ODFs by the solvent-casting technique. Paracetamol and ketoprofen were utilized as model drugs to establish the highest loading capacity of the designed films. All obtained formulations exhibited optimal plasticity, mechanical features, and disintegration time. Additionally, the high drug loading, 70% (*w/w*) of film weight, was achieved [79]. An example of a commercially available drug dosage formulated with using hydrophobic polymer is Niquitin Mint Oral Film Strips based on Eudragit® L 30 [80].

## 4. Overview of ODF Formulations Available on the Pharmaceutical Market

ODFs continue to be a current concern for technologists and researchers, who concentrate on their continuous development. The pharmaceutical market is increasingly abundant with the availability of a variety of ODFs. The instances of commercially available ODFs and excipients utilized in their manufacturing are given in Table 5.

## 5. Conclusions

Polymers are regarded as essential excipients in the pharmaceutical technology, having a significant influence on the advancement of ODFs. Concerning the properties of the polymeric material, ODFs disintegrate or dissolve spontaneously in the mouth, which results in the formation of a solution, soft paste, or suspension without the necessity for fluids’ intake. When designing ODFs, attention should be paid to the proper selection of polymers. The overall purpose is to implement cost-efficient, biocompatible, versatile, non-toxic polymers. Among different types of polymers, their blends are usually used so that they can mutually complement their properties, as polymeric materials of lower molecular weight disintegrate faster, while using higher molecular weight polymers provides ODFs with improved mechanical features. In this regard, it is essential to ensure the right balance between the mechanical characteristics and the disintegration time demanded for ODFs. The multitude of polymeric materials’ types, different polymer classes, and various polymer-to-polymer ratios result in an explosive amount of different formulations and a broad spectrum of ODFs’ specifications. Consequently, it is extremely important to have a thorough comprehension of the system under development to prevent obtaining dosage forms with undesirable properties. This manuscript summarizes the role of polymers alone or in combination and their impact on the designed formulation and provides insight into their functionality in the process of ODFs’ manufacturing.

## Figures and Tables

**Table 1:** Properties of cellulose derivatives depending on their functional groups [5,9,15,16,18,19,20].

Cellulose Derivative	Manufacturing Metod	Characteristic
Methylcellulose (MC)	Produced in the reaction of methyl chloride and a cellulose activated by mercerizing the cellulose with aqueous alkali in the presence of a C_2_-C_3_ alkyl chloride as a reaction medium at a temperature from 65 °C to 90 °C and at pressure from 3 to 15 bar.	Methyl ether of cellulose, in which the content of methoxyl groups is 27–32%. It takes the form of white, light-yellow, or light-gray powder or granulate. It does not dissolve in hot water, but swells in cold water and forms dispersions. The degree of substitution (DS) influences the solubility and viscosity of the polymer. The viscosity of the 2% (*w/w*) aqueous solution ranges from 5 to 75,000 mPa⋅s. DS is determined as the average level of substituted hydroxyl groups per glucose unit in cellulose molecule. Its value is in the range from 0 to 3.
Carboxymethyl- cellulose sodium salt (NaCMC)	Synthesized by the alkali-catalyzed reaction of cellulose with monochloroacetic acid or its sodium salt under alkaline conditions in the presence of an organic solvent. Hydroxyl groups replaced by sodium carboxymethyl groups in C2, C3 and C6 of glucose, the substitution being slightly predominant at the C2 position.	Semisynthetic, linear, anionic derivative of cellulose, known as crosscarmellose sodium. White, odorless powder of molecular weight 90,000–700,000. Readily dispersed in water, forming a transparent or colloidal solution. One percent viscosity of the aqueous solution ranges from 5–13,000 mPa⋅s. NaCMC plays a significant role as a thickener and is suitable for applications requiring a rapidly soluble base. DS value of NaCMC has a significant effect on the properties of membrane-forming solutions because higher DS values are closely associated with reduced inter-chain interactions considering the increased substitution of hydroxyl sites. NaCMC has been found to be a valuable excipient for the development of ODFs with good transparency and capacity for a broad array of drugs. NaCMC-based films are also characterized by the ability to carry a broad spectrum of DS and good compatibility with starch to form single-phase polymeric matrix films with enhanced mechanical or barrier characteristics.
Hydroxyethyl- cellulose (HEC)	Obtained by introduction of ammonia-activated cellulose into water sodium hydroxide solution, which is responsible for its swelling, then degassed and mixed with an appropriate amount of III-row butanol. The processes take place in the presence of nitrogen and the introduction of ethylene oxide. The whole is neutralized by acetic acid and dried.	Fully fragranceless, flavorless, and non-toxic white to pale-yellow powder that is easily dissolved in hot and cold water and insoluble in organic solvents. After dissolution in water it creates a transparent, viscous solution. There are varieties available with different degrees of substitution, with 2% water solutions varying in viscosity from 2 to 20,000 mPa⋅s. The substance is stable at pH 2–12.
Hydroxypropyl cellulose (HPC)	Formed by the connection of propylene oxide to -OH groups in cellulose.	White- to slightly yellow-colored, odorless, inert, and tasteless powder. The viscosity of 2% of water solutions is from 2 to 6500 mPa⋅s. It is well soluble in water at temperatures below 38 °C, precipitating at temperatures > 40 °C. Solutions are stable at pH 6–8. HPC dissolves freely in cold water and provides a smooth, transparent, colloidal solution. It is insoluble in hot water and forms a flocs. HPC is used as a film-forming material due to its good pharmaceutical and mechanical properties. HPC films exhibit good loading capacity, transparency, and balanced bioadhesive performance associated with the swelling ability.
Hydroxypropylmethyl cellulose (HPMC)	Obtained by alkylation of cellulose with methyl chloride or a combination of methyl chloride and propylene oxide.	Soluble in water (below 60 °C) and in organic solvents. White, creamy, odorless, and tasteless powder. MW ranges from 10,000 to 1,500,000. The viscosity of 2% aqueous solution is from 2 to 200,000 mPa⋅s. Stable at pH 3–11. The use of HPMC is beneficial in increasing the solubility and dissolution of drugs with poor water solubility.

**Table 2:** Methoxy and hydroxypropoxy groups’ content in the various types of HPMC [13].

Substitution Type	Methoxy (%)		Hydroxypropoxy (%)	
	Min.	Max.	Min.	Max.
1828	16.5	20.0	23.0	32.0
2208	19.0	24.0	4.0	12.0
2906	27.0	30.0	4.0	7.5
2910	28.0	30.0	7.0	12.0

**Table 3:** Examples of ODFs made on laboratory-scale based on cellulose derivatives.

Polymers	Other Excipients	Drug	Manufacturing Method	Comments/Observations
HPMC E3, E5, E15	xantan gum, xylitol, glycerin, propylene glycol	triclosan	solvent casting	Placebo films were created from HPMC E3, E5, E15, but the most desired properties were shown for type E5 (at concentration 2.2%, *w/v*). Of the different film modifiers used (xanthan and guar gums, carrageen), xanthan gum exhibited the finest capacity to enhance the film-forming properties of HPMC. The addition of mannitol to the formulation resulted in opaque films, while films containing xylitol and sorbitol gave transparent product. Nevertheless, for film creation the preffered one was xylitol in regard to its more negative solution heat and lower hygroscopicity in comparison to sorbitol. It is expected that agents with a more negative solution heat would give a stronger cooling sensation in the mouth, which is important in taste-masking effect [43].
pullulan, HPMC E5, HPMC E15	PEG	granisetron hydrochloride	solvent casting	ODFs were developed from 2.5%, 5%, 7.5%, and 10% solutions of pullulan, HPMC E5, or HPMC E15. Pulluan and HPMC E15 showed the desired film-forming capacity while ODFs made of HPMC E5 possessed average film-forming properties and were semitransparent. An examination of the release of the drug and mechanical features revealed that for ODFs made of 5% pullulan granisetron release rate was the highest, about 92% of the amount of drug was released, and simultaneously the fastest [44].
HPMC E5, HPC, PVA, SA	sucralose	anastrazole	solvent casting	Among the examined formulations, films obtained utilizing HPMC E5 showed shorter disintegration time (15 s) with satisfactory mechanical properties [45].
HPMC E3	glycerol 85%, carbomer 974	influenza vaccine	solvent casting	Formulation of the proposed combination was advantageous as it provided improved stability for influenza vaccine and constituted a suitable, inert carrier for the drug [46].
HPMC (Metolose 60SH-50)—10%, mesoporous colloidal silica—10%.	glycerol	haloperidol	solvent evaporation pour-off method	Haloperidol solution was printed on ODFs in the form of a QR code [47].
HPMC	PEG 400, Na CMC	escitalopram	solvent casting	Different concentrations of HPMC were tested: 5%, 7.5%, 10%. The thickness of the formulations with polymer concentration 5% was assessed to be superior compared to formulations containing 7.5% and 10%. The viscosity of the solutions with higher polymer concentration was too high and the presence of lumps in the solution created a great challenge to spread [48].
Pharmacoat 606	glycerol 85%, PEG	loratadine hydrochloride	solvent casting	Disintegration time was mainly influenced by the polymer concentration (2.5%, 5%, or 10%): With the increased amount of polymer, increased disintegration time was observed. The shortest disintegration time was noted in formulations containing HPMC at concentration of 2.5%. ODFs containing glycerol as plasticizer possessed better mechanical strength compared to formulations with polyethylene glycol. The films containing polyethylene addition were characterized by greater moisture content and tended to stick together. Moreover, volunteers claimed that films with PEG or polyethylene glycol/glycerol blend were of unsavory taste. It was decided to use glycerol at a concentration of 2.5% to strike a balance between mechanical properties and taste-masking effect. Interestingly, a mixture of both plasticizers did not improve the mechanical properties of ODFs [17].
HPMC E15	pregelatinized starch, maltitol, sucralose	levocetirizine hydrochloride	semisolid extrusion 3D printer	The polymer was applied at concentration of 17% (choosen experimentally from 15%, 16%, 17%, 18%, 20% in terms of viscosity of solution that would be printed in a satisfactory manner). The resulting formulations showed optimal elasticity and rapid drug release in vitro by complete dissolution below 2 min [49].
HPC:HPMC in 1:4 ratio; dextran maltodextrin	PEG 400, glycerol 85%, sorbitol, microcrystalline cellulose, CMC	amphotericin B	solvent casting	The optimised ODF composition consisted of 1% drug, 25% dextran, 25% maltodextrin, 5% sorbitol, 10% microcrystalline cellulose, 10% PEG 400, 10% glycerol, 3% HPM, and 12% HPMC. The formulation disintegrated quickly (60 s), providing quick release (>80% in 10 min) in artificial saliva [50].

**Table 4:** Utilization of sodium alginate and chitosan in the ODFs’ formulation.

Polymer	Characteristic	Utilization in ODFs
Sodium alginate	Alginates are naturally occurring polysaccharides extensively utilized in the pharmaceutical industry because of their broad availability, safety, and biocompatibility They are sourced from various genera of brown algae (mainly *Laminaria hyperborea, Macrocystis pyrifera, Ascophyllum nodosum* and in lesser extent *from Laminaria digitate, Laminaria japonica, Eclonia maxima, Lesonia nigrescens, Sargassum* sp.) or synthesized by some bacteria such as *Azotobacter vinelandii* or mucoid strains of *Pseudomonas aeruginosae.* The bacterial alginates possess O-acetyl groups, whereas these are not contained in the structure of algal alginates. Moreover, bacterial alginates are characterized by higher molecular weights in comparison to the algal ones [18].	Sodium alginate- and PVA-based ODFs with sildenafil citrate obtained by solvent-casting method were successfully developed and characterized by short disintegration time (20 s) [64]. Sodium alginate-, pectin-, and gelatin-based ODFs with zolmitriptan and propylene glycol as plasticizer were developed by solvent-casting method. According to the physical and mechanical properties, sodium alginate was selected as the best film former [65].
Chitosan	The polymer is obtained by the deacetylation of chitin from crustacean shells, which consist of β-(1-4)-2-acetamido-D-glucose and β-(1-4)-2-amino-D-glucose units. This biodegradable polysaccharide possesses satisfying film-forming properties. Chitosan-based films show good permeability, biocompatibility, and advantageous pharmacological profile (antibacterial and antifungal properties of chitosan *per se*) [18,61,63].	ODFs with donepezil formulated from chitosan blended with polyester were characterized by fast disintegration time and optimal drug release [66]. Chitosan and pullulan electrospun orodispersible films containing acetylsalicylic acid were developed with the total polymer content kept at 10% (*w/v*). The weight ratios of chitosan to pullulan in variously mixed blend solutions were 0:100, 10:90, 20:80, 30:70, 40:60, 50:50, and 60:40, accordingly. Since electrospinning of pure chitosan is troublesome, owing to its intra-molecular interactions, polycationic nature in solution, and rigid structure, pullulan was utilized to facilitate the electrospinnability of the solutions by raising viscosity and decreasing conductivity and superficial tension [67].

**Table 5:** Exemplary ODFs accessible as OTC or Rx preparations.

Product	Polymer(s)	Additional Excipients	References
*Drug*			
Benadryl Allergy quick dissolve strip *Diphenhydramine*	carragen, pullulan	glycerin, propylene glycol, acesulfam K	[81]
Chloraseptic Sore Throat Relief Strips *Benzocaine*	corn starch	erithrytol, malic acid, menthol, macrogol, sucralose	[82]
D-Fusion Film 800 UI *Cholecalciferol*	pullulan, HPC	xylitol, sucralose, glycerol	[83]
Exservan Oral film *Riluzole*	HPC	glycerol, glycerol monooleate, polacrilex resin, sucralose, xanthan gum, xylitol	[84]
Gas-X Thin Strips *Simethicone*	maltodextrin, HPMC	macrogol, sorbitol, sucralose	[85]
IvyFilm, IvyFilm Kiddies *Hedera helix extract*	Pullulan	glycerol, sucralose	[86]
Listerine PocketPaks Oral Care Strips *Menthol*	pullulan, carragenan, xanthan gum	menthol, aspartame, acesulfam K, macrogol	[87]
Niquitin Mint Oral Film Strips *Nicotine*	methacrylic acid—ethyl acrylate copolymer (1:1)	sucralose, sodium hydrogen carbonate, peppermint flavor	[80]
Pedia-Lax Quick Dissolve Strip *Sennosides*	HPMC	malic acid, glycerol, menthol sucralose	[88]
Sudafed PE *Phenylephrine*	maltodextrin, pullulan, carrageen	acesulfam K, aspartame, glycerin, macrogol	[89]
SildeHEXAL SF orodispersible film *Sildenafil*	HPMC	glycerol, sucralose, peppermint flavor, levomenthol	[90]
Sildenafil IBSA Orodispersible film *Sildenafil*	Maltodextrin	glycerol, polysorbat 20, propylene glycol, lemon and grapefruit flavors	[91]
Tusheel *Hedera helix extract*	pullulan, NaCM	glycerol, sucralose,	[92]
Setofilm *Ondansetron*	PVA	PVA, macrogol, levomenthol, glycerol	[93]
Sympazan oral film *Clobazam*	HPC	citric acid, glycerol monooleate, maltiotol	[94]
Zentrip *Meclizine hydrochloride*	HPMC	acesulfame potassium, mannitol, menthol, orange oil, polyethylene glycol 400, sucralose	[95]
Zuplenz *Ondansetron*	HPMC	erythrytol, sucralose, peppermint	[96]
